# Strategies for automating analytical and bioanalytical laboratories

**DOI:** 10.1007/s00216-023-04727-2

**Published:** 2023-05-13

**Authors:** Kerstin Thurow

**Affiliations:** Center for Life Science Automation, University of Rostock, Rostock, Germany

**Keywords:** Laboratory automation, Analytical chemistry, Robotics, Automation systems, Automation strategies, Systems engineering

## Abstract

Analytical measurement methods are used in different areas of production and quality control, diagnostics, environmental monitoring, or in research applications. If direct *inline* or *online* measurement methods are not possible, the samples taken have to be processed *offline* in the manual laboratory. Automated processes are increasingly being used to enhance throughput and improve the quality of results. In contrast to bioscreening, the degree of automation in (bio)analytical laboratories is still low. This is due in particular to the complexity of the processes, the required process conditions, and the complex matrices of the samples. The requirements of the process to be automated itself and numerous other parameters influence the selection of a suitable automation concept. Different automation strategies can be used to automate (bio)analytical processes. Classically, liquid handler-based systems are used. For more complex processes, systems with central robots are used to transport samples and labware. With the development of new collaborative robots, there will also be the possibility of distributed automation systems in the future, which will enable even more flexible automation and use of all subsystems. The complexity of the systems increases with the complexity of the processes to be automated.

**Graphical Abstract:**

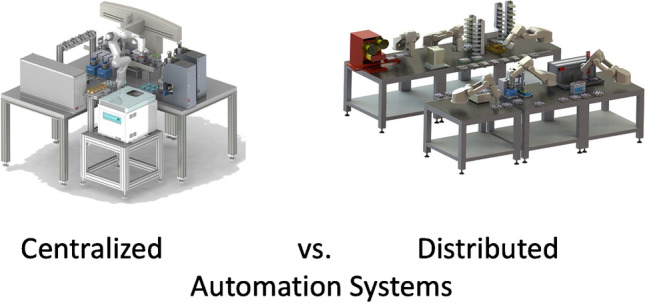

## Introduction

### Automation in life sciences

Automation has meanwhile covered many areas of industrial production. The benefits of automation are obvious. Automated processes can be more precise than humans, and they can be used for many dangerous and repetitive jobs. The quality of produced goods can be equalized. Human influences on the quality of processes can be eliminated. Automated systems can work more efficiently and cost-effectively. This makes it possible to keep jobs in Germany or even bring them back to Germany. An increasingly important aspect is the growing shortage of skilled workers, which can only be solved with increasing automation.

All the advantages mentioned also apply to the automation of laboratory processes. Despite this, the degree of automation in laboratories, particularly analytical and bioanalytical laboratories, is still far behind the level of automation found in industry.

Automation in the life sciences has long been dominated by the pharmaceutical industry. The need to handle large numbers of samples in the development of new active ingredients arose early on. From this, the methodology of high-throughput screening (HTS) was developed, which is used in pharmaceutical research to carry out biochemical, genetic, or pharmacological investigations of millions of compounds. Numerous drugs have been developed using HTS processes, such as the cancer drugs gefitinib (AstraZeneca) [1] or lapatinib (GlaxoSmithKline) [2], the HIV drugs tipranavir (Boehringer Ingelheim) [3] and etravirine (Tibotec Pharmaceuticals) [4], or the diabetes drug sitagliptin (Merck) [5]. The development of antiviral drugs by screening compound libraries using a cell-based phenotypic assay has also been described. Twenty-five of the 45 hits identified could be validated in subsequent studies [6]. A classic example of HTS applications is also the Tox21 initiative, which aimed to determine the toxicity of more than 10,000 environmentally relevant compounds [7]. In addition to a central robot, the system developed for this purpose also included incubators, dispensers for dosing in 1536 MTP and fluorescence or UV readers [8].

Meanwhile, there is also an increasing need for automation for classic laboratory applications in medical diagnostics, environmental analysis, quality, or process control. The reasons for this are the increasing number of samples and new diagnostic methods and increasing requirements in the field of quality control but also an increasing shortage of skilled workers [9]. The COVID-19 pandemic recently has been accompanied by an enormous need for analytical investigations, which has led to the development of a wide variety of automation systems [10].

### Processes in (bio)analytical chemistry

The metrological determination of substances and components plays an important role in numerous areas. Analytical processes can be extremely diverse and have different requirements depending on the area of application. A large area of application for analytical measurements is the field of quality control, e.g., in the pharmaceutical or food industry. Due to increasing regulations in a wide variety of applications, the number of samples in this area is increasing sharply. Quality controls are often carried out during production and must then also have correspondingly short analysis times. Another field of application is medical diagnostics. Here, human samples such as blood, urine, or tissue are tested for a wide variety of substances such as vitamins, drug residues, biomarkers, and others more. However, the matrix load is quite high in these applications. Numerous substances have to be separated from the analytes that are actually to be determined in complex sample preparation steps. The same applies to environmental analysis, where matrices such as soil, water, sludge, or waste are to be analyzed using measurements. But also in the academic field, in research and development, extensive analytical investigations have to be carried out in various areas. Great variability often characterizes these processes.

Analytical and bioanalytical processes differ significantly from classic bioscreening processes. “To screen” means to select, to raster, or to filter. The aim of screening methods is therefore to provide an overview or a rough classification. The speed of answering questions is of great importance in screening methods, e.g., to examine numerous samples, to achieve a high-throughput (as in the pharmaceutical industry) or to obtain individual results rapidly (as with rapid tests). Losses in sensitivity are accepted in the process. In contrast to this, the aim of analytical methods lies in the precise answering of questions. More importance is attached to high sensitivity and selectivity. For this reason, the analytical methods used are more complex and usually require more extensive preparation of the sample material before the actual analytical measurement. The time requirement of the method may be higher.

Screening methods usually consist of a few process steps such as dosing, incubation, and analytical determination. Simple systems such as photometers, UV, or fluorescence readers are used here. In general, the samples are processed in parallel in the microtiter plate format. In addition, very moderate environmental conditions (temperature, pressure) and predominantly aqueous solutions are used. The processes are usually very similar, so that different screening processes can be carried out on one automation system without major system adjustments. In contrast, (bio)analytical methods are more complex in nature. The sample matrices range from human material such as blood, serum, plasma, urine, or saliva to environmental samples (water, mud, soil etc.), food samples, and complex samples for industrial quality control.

The isolation of the target substances to be determined can contain numerous process steps. To lower the salt, protein, or phospholipid concentrations in a sample, dilutions are used, among other things, but the concentration of the target substances is also reduced. This “dilute and shoot approach” is often used in bioanalysis for urine samples [11]. Filtrations are often used in biological matrices to remove cellular components or proteins. In automation, filters in microtiter plate format are used, which leads to a further increase in sample throughput [12]. Centrifugation is used, for example, to separate cellular and non-cellular blood components to obtain plasma or serum. Protein precipitation is a quick and easy method to remove proteins from biological matrices such as plasma or serum samples by adding a precipitating reagent [13]. Organic solvents such as acetonitrile or methanol lower the dielectric constant of the protein solution. The additional reduction in hydrophobic interactions by displacement of water molecules increases the electrostatic interactions between the protein molecules, which leads to irreversible precipitation of the proteins. Extractions serve to select the target substances in the purest possible form. Here, the solid-phase extraction is of particular importance and is now one of the most frequently used sample preparation methods in bioanalysis [14]. The main advantages are the high selectivity, the comparatively simple automation [15, 16], and possibilities for the simultaneous concentration and adaptation of the solvent to the mobile phase as well as the low solvent requirement. In addition, numerous formats are available.

While the actual analytical procedures are highly automated today, the samples are usually still prepared manually. For automation, suitable processes must be developed that can carry out the above process steps fully automatically with high reliability and precision.

## Automation strategies

### Introduction

Analytical processes can be automated in different ways. In the simplest case, the desired parameters of a sample can be determined by direct measurement. With these *inline* measurement methods, the substances of interest are determined using spectroscopic methods or the use of electrochemical sensors, among other things. The measuring system is integrated into the production line, and the measurement takes place in real time.

If a direct measurement is not possible, e.g., due to the measuring method used, a sample must be taken before the actual metrological determination. The qualitative determination and quantification of the samples of interest thus take place outside the actual production process. If the measuring system is connected directly to the process via a sampling device, this is referred to as an *online* measuring method. *Online* measurement technology enables a quasi-real-time determination of the parameters of interest.

Both methods are used in process analytical technology (PAT) and are characterized by a high degree of automation.

The PAT measurement methods are often inadequate for more precise determinations of individual parameters. More complex methods are used here, which often require extensive processing of the samples before the actual metrological determination. *Offline* measurement technology is performed outside the process and is used to analyze the end product or the process data after production. *Offlin*e measurement technology can be more accurate and specific as the samples are analyzed under controlled conditions.

The division into *inline/online* methods comes from the field of PAT and is not readily applicable to many application areas of (bio)analytical measurement technology. In contrast to classic PAT, there are numerous applications that require the processing of individual samples. This applies, among other things, to medical applications in which patient-specific samples have to be examined for certain components. But also in the field of environmental monitoring or in the quality control of food, individual samples are taken at different control points and have to be examined for ingredients. Here, we are always in the area of *offline* analysis, unless specific biosensors are used, the use of which is limited to a few applications. *Offline* analysis, especially in the areas of sample preparation, has so far only been slightly automated and requires appropriate automation concepts.

### Automation strategies: overview

Different parts of an automation system are required for the automation of (bio)analytical processes. In principle, a distinction can be made between data generation and data handling. In addition to the actual measuring system, the area of data generation contains all other subsystems that are required to finally generate analytical measurement data. According to the areas of sample preparation mentioned in the “Processes in (bio)analytical chemistry” section, these can be systems for handling samples (e.g., dosing) and manipulating samples (e.g., heating, shaking, incubating, grinding, purification). The scope of the required subsystems depends on the respective process. In addition, procedures are required for complete automation that take over the transport of the samples and the labware used between the individual subsystems. Conveyor belts or robots are used for this.

The second area to be automated is data handling. First, the actual processing of the measurement data collected to generate secondary data, analysis reports, etc. should be mentioned here. However, the connection of the automation systems to higher-level systems such as LIMS, ERP, or corporate-specific in-house workflow systems must also be considered. Particular attention is paid to the agreement of interfaces for data exchange and data formats between different software solutions.

**Fig. 1:**
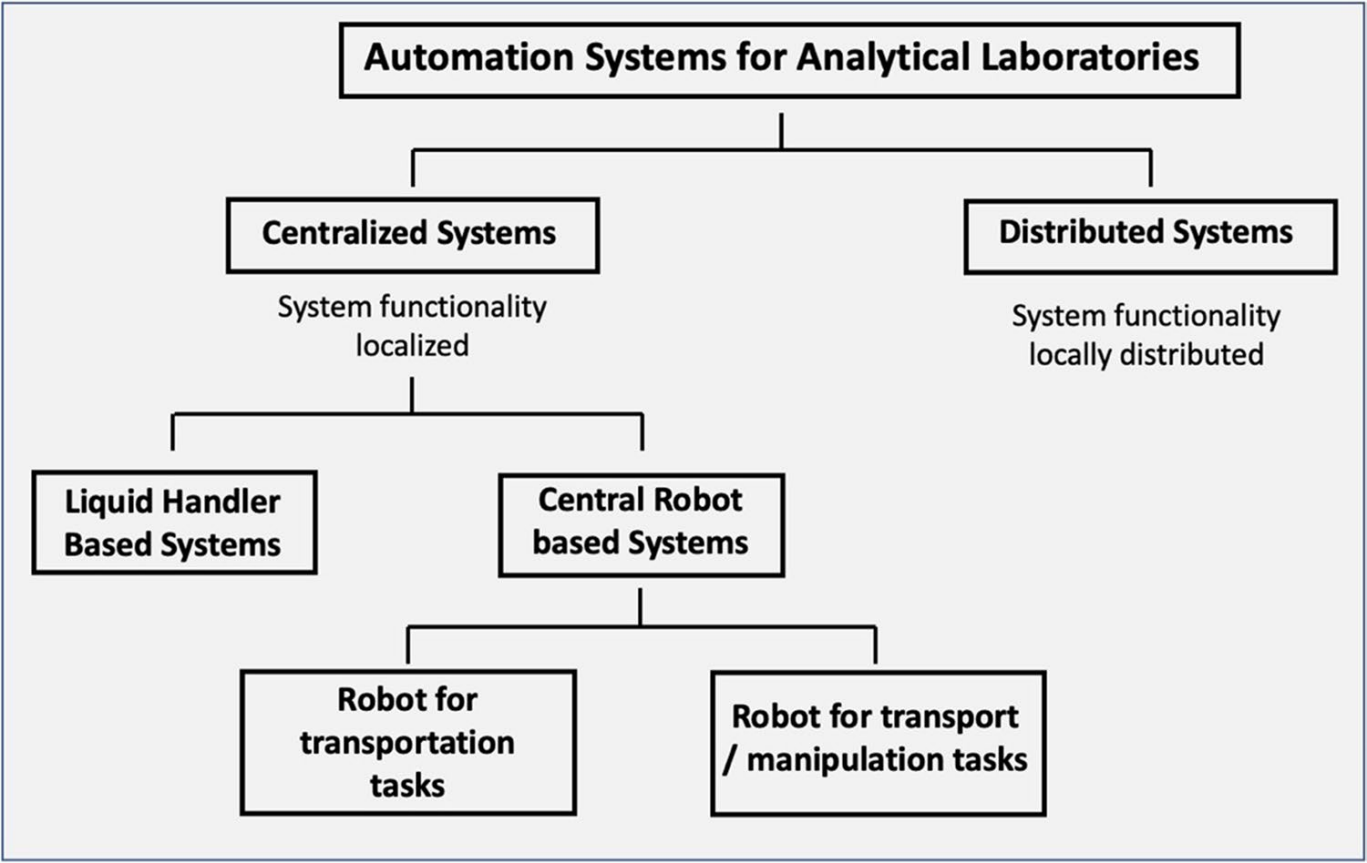
General strategies for automating analytical and bioanalytical laboratories

Different strategies are conceivable for the automation of analytical and bioanalytical processes. Liquid handler-based systems represent the simplest form. They enable highly parallel processing of samples but are usually limited to pure liquid handling processes. The integration of peripheral devices is only possible to a limited extent. Fully automated systems based on a central robot can be used for analytical methods with extensive sub-process steps. The central robot can either only be used as a transport instance for the transport of samples and labware between the integrated substations or it can also handle the manipulation of samples itself (see Fig. 1).

Both approaches can be designed as closed or open systems [17]. Closed systems are designed and optimized for a specific application. As a result, they can work highly efficiently, achieve high-throughput, and are quite inexpensive. Process changes or even the establishment of new processes is associated with a great deal of effort on such systems; the integration of additional or other devices is usually not possible. Open systems, on the other hand, offer greater flexibility. Depending on their equipment, they are designed more for a specific process group. The change of processes as well as the establishment of new processes are possible. New devices can be integrated into the systems and thus increase the range of functions and the type and number of processes to be automated. However, this flexibility is accompanied by significantly higher costs. In addition, the achievable throughput is usually lower than with optimized proprietary systems.

Recent developments in robotics increasingly enable the use of distributed system strategies. Here, processes are no longer processed centrally in one place, but various process-relevant substations are provided with robotic components.

### Liquid handler-based systems

**Fig. 2:**
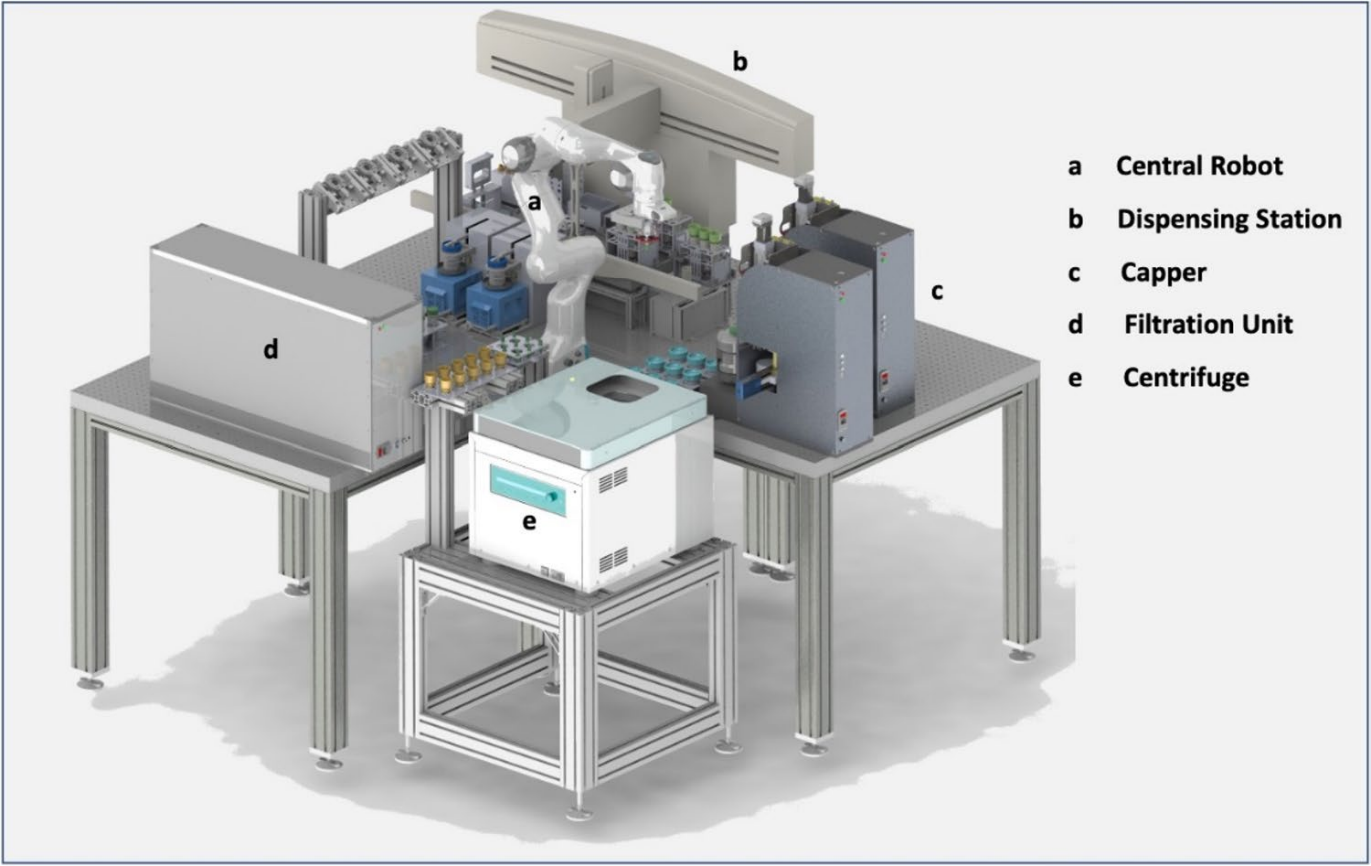
Complex robotic system for cell-based medical diagnostics

Liquid handler-based systems represent the simplest form of automation. They are based on Cartesian robots and are primarily designed for dosing liquids. Automated liquid handling systems can have a different number of channels. Depending on the equipment, three main variants can be distinguished—single-channel systems, systems with 1–8 channels and highly parallel systems with more than 8 and up to 1,536 channels (see Fig. 2). As the use of micrtiter plates increased, automated liquid handlers were developed with an appropriate number of channels for the most common plate formats 96, 384, and 1.536. The channels in the pipette heads have a permanent spacing that corresponds to the spacing of the wells in the microtiter plates. The systems thus enable the parallel processing of numerous samples. Liquid handlers are optimized for handling samples in microtiter plates and are therefore particularly suitable for processing samples that can be handled in MTP. Individual samples would therefore have to be reformatted accordingly before processing. Alternatively, the labware can be adapted to handle individual samples. The SBS external dimensions should be retained to enable easy processing on the liquid handler. If individual vessels are used that do not correspond to the standard format of MTPs (96 samples in a grid of 8 × 12), multichannel pipettors can hardly be used. The so-called Span-8 functionality is ideal here, with which up to 8 channels can be used individually and at freely definable distances from one another for the dosing processes.

Liquid reservoirs are to be provided on the deck of the liquid handler for the dosing processes. Usually, reservoirs in MTP format are used here, which, depending on the pipetting head, can be used as full reservoirs (for 96 and 384 heads, one solvent possible) or as half or quarter reservoirs (for 8-channel systems, 2–4 different solutions possible). The use of self-filling reservoirs limits the number of positions that must be provided on the liquid handler deck for the provision of the solvent.

Liquid handlers can be equipped with additional devices that extend the functionality of the systems. Usually, these are shakers, heaters, and coolers but also systems for automated sample purification such as solid-phase extractions. Centrifuges, incubators, analytical measurement systems, etc. can also be integrated, provided there is sufficient space on the liquid handler deck and the devices to be integrated can be equipped by the liquid handler. In addition to the pipetting head, liquid handlers with such an extended range of functions then have a second or even a third arm with a special gripper, which transports samples and labware to and between the different substations.

Liquid handler-based systems have been described for the fully automated determination of vitamin D in blood samples, among other things [18]. The heart of the system is a liquid handler with 8 parallel, freely configurable channels for dosing the liquids and a second arm that is used with a gripper to transport the labware. The system enables the proteins to be separated using an integrated centrifuge. The purification is carried out by an integrated fully automatic solid-phase extraction. Furthermore, shakers and incubators as well as refillable reservoirs and special racks for the provision of the original samples in Eppendorf vials are integrated on the deck. The overall system enables the processing of up to 96 samples. A further increase in the number of samples (up to 288) can be achieved by using special phospholipid removal cartridges, since the centrifugation of the samples can be omitted. The system can also be used flexibly for other bioanalytical methods such as the determination of THC and its derivatives in serum, saliva, and urine [19] or the determination of benzodiazepines. Systems with automated solid-phase extraction have also been used to determine diuretics in doping control [20] or to detect beta blockers in blood [21].

### Systems with central robot as transport instance

Another variant of automation is complex systems, the center of which is a central robot. The devices and systems required for processing the sub-steps are arranged in the work area of the central robot, which transports samples and labware between the different stations. Liquid handlers are usually used to implement the dosing processes, or in the case of larger volumes to be dosed, dosing pumps are used. The number of peripheral devices to be integrated depends on the range of the central robot. The peripheral devices should have suitable interfaces for system internal communication and allow a robot to access the device. For example, centrifuges should be equipped with a lid that opens automatically for the introduction and removal of samples. Autosamplers of connected devices should be designed in such a way that the gripper of a robot can also feed and remove samples. If this is not the case, adjustments to the hardware may be necessary. Depending on the design of the gripper, different formats can be handled—from microtiter plates to individual sample vessels of different sizes. If different formats are to be processed within a method, it may be necessary to change the gripper. Alternatively, universal grippers can be used that cover a large range of labware dimensions. The labware can, but does not have to, be executed in SBS-MTP format. Systems of this type allow single sample handling and are therefore suitable for processes that cannot be reformatted to the MTP format. Depending on the process, the development of special racks or even additional systems may be necessary. The rate-limiting step in such systems is the instrument with the longest processing time of the samples.

This system concept usually requires adjustments to the existing standard operating procedures, as other devices/systems are used for individual sub-steps. For example, pipetting is done manually with classic manual single- or multichannel pipettes, which cannot usually be handled with a robot. Instead, automatic dispensers or liquid handlers are used. Complete 1:1 automation, i.e., the identical translation of a manual process to an automation system, is therefore usually not possible.

Complex, fully automatic systems have been described for different applications. Tsina described a system for an automated HPLC method to detect mycophenolic acid in human plasma [22]. In addition to a laboratory robot, the system has various stations for sample preparation, such as stations for weighing, diluting, dispensing, and pipetting. Furthermore, two online HPLC systems with optical detectors were integrated. A fully automatic, robot-based system for sample preparation and analysis was also established for routine measurements in the quality control of active ingredients and pharmaceutical end products [23]. In addition to several robotic components for sample transport, the system also has stations for homogenization, temperature control, dispensing, and pH measurement. Another system with a central robot was developed for cell-based medical diagnostics, which enables the fully automatic processing of sputum samples for subsequent examination using cell CT (see Fig. 2). The system has an integrated centrifuge, a liquid handling system for the realization of the dosing steps in the µL to mL range, specially developed cappers, adapted vortexers, and a filtration unit [24]. In the process, the height of the resulting cell pellets must be detected several times, which is realized using image processing methods [25].

### Systems with central robot as transport and manipulation instance

An extension of the concept mentioned above are systems with central robots which, in addition to transporting samples and labware, can also take on direct manipulation steps. If dual-arm robots are used here, a process analogous to manual processes is possible. Manually used devices such as pipettes or syringes can be integrated into the systems. This means that actual 1:1 automation is possible; no changes to existing standard operating procedures are required. The speed of the overall system is determined by the central robot and depends on the times required to carry out individual process steps. Automation systems with dual-arm robots were used for different applications. Chu developed a corresponding system for bioanalytical applications. The dual-arm robot used enables the complete processing of samples including pipetting steps, the opening, and closing of individual vessels, the transfer of samples to devices for sample purification (ultrasonic device, solid-phase extraction, shaker, heater, etc.), and the final positioning of the prepared samples in connected analyzers [26]. The system was used, among other things, for the determination of cholesterol in biliary endoprosthesis using gas chromatography-mass spectrometry (GC–MS) [27] and for chirality studies [28]. Dual-arm robot-based systems have also been described for automated downstream analysis of epidermal models [29] or anticancer drug compounding [30].

### Distributed robotic systems

Depending on the application and level of equipment, central automation systems can become huge. The associated space requirement is sometimes not available in laboratories. In addition, devices and components are tied into complex automation systems and are not available for other processes, even if they are not required in a current process flow.

With the development of lightweight robots (also known as collaborative robots, cobots), more cost-effective variants for automation are now available. These robots are characterized by a lighter construction, low speeds, and additional sensors. As a result, there is no need to install additional safety precautions (light curtains, housings, …) and the robots can work in proximity to people and even share the same workspace [31].

**Fig. 3:**
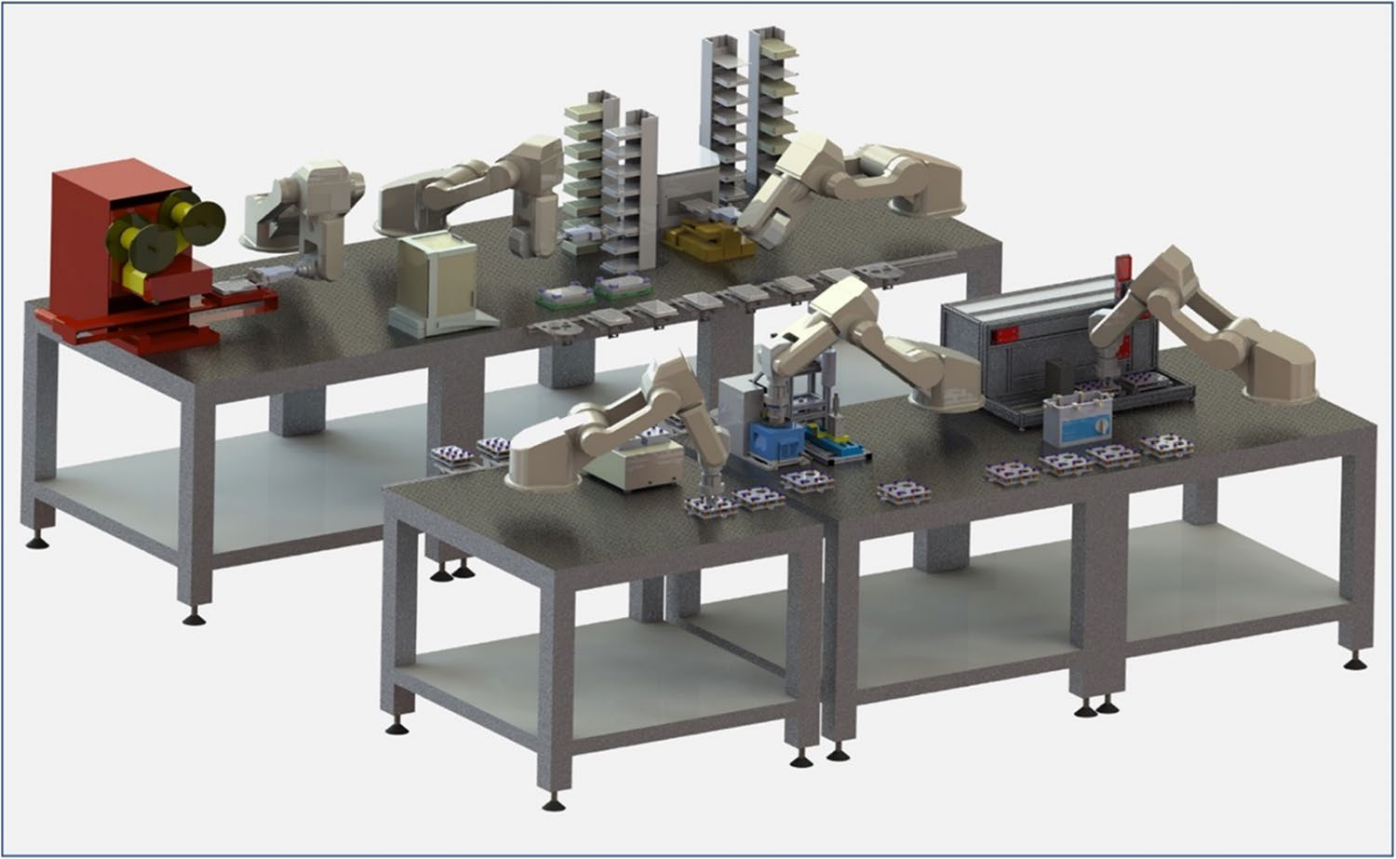
Distributed automation system

The lightweight robots can be integrated into the above-mentioned central systems as central robots or used to equip individual systems and system groups. Existing device technology can be used and automated through integration with a robot. However, as with the central systems, there may be a need to adapt the devices to robotic operation. Existing spatial constellations can be used. This creates distributed automation systems (see Fig. 3). The individual stations can be combined with each other in any form in complex processes or used individually. This leads to a high degree of flexibility in the processing as well as in the utilization of the existing devices. Such a distributed system is primarily suitable for open system structures, since it offers the possibility of any expansion of the overall system. With this approach, the investment required depends on the number of devices to be equipped with a robotic component.

## Discussion

### Advantages and disadvantages of automating (bio)analytical laboratories

The automation of laboratory processes has numerous advantages. The sample throughput can be increased without additional staff costs, especially when the automation systems are used to full capacity, and can be adapted to the increased requirements in many areas for the number of samples to be processed. Sample processing times can be shortened, and thus, results can be made available more quickly, especially for critical processes in medical diagnostics or in process monitoring. Operating and testing costs can be reduced, and services can be offered at a competitive level. A reduction in the personnel costs as a proportion of the total costs is possible in laboratories by increasing the degree of automation from approximately 15% to up to 4% [32, 33]. Risks from human operators are minimized, and occupational safety, especially when working with dangerous or infectious materials, is significantly increased. The use of the available analytical systems is optimized, which in turn is associated with increased efficiency and reduced costs. A critical aspect is also the high traceability of samples in automation systems through the monitoring of the individual process steps. A major advantage of automation is the standardization of process flows through the use of identical measuring systems (metrological twins). Metrological twins are increasingly used in scientific research, quality control, and other applications where precise and accurate measurements are essential. By using identical instruments, users can be confident that any differences in measurement results are due to the properties of the sample being measured, rather than variations in the instruments themselves. This helps to improve the accuracy and reliability of experimental results and reduces the risk of measurement errors.

### Factors influencing the selection of a suitable automation concept

The selection of a suitable strategy for automating (bio)analytical processes depends on numerous parameters. First, it is essential to identify and evaluate the process to be automated, including all the necessary sub-steps. A detailed analysis of all sub-processes as well as the respective parameters and boundary conditions forms the basis for the development of a suitable automation concept. The decisive factor is the type of sample to be examined, as this determines the sub-steps required for the preparation of the samples. If the samples can be handled in MTP format and large numbers of samples are to be processed in parallel with low volumes up to 1 mL, liquid handler-based systems with multichannel heads are ideal. If single sample handling is required, a decision must be made whether parallelization in MTP format (but still with single sample containers) is possible. The question of the sample containers also determines to a large extent the dimensions that have to be considered for robotic grippers. Furthermore, questions about the number of samples to be processed and the desired throughput must be answered. Another factor is the required environmental conditions for the respective process. If there are specific requirements for temperature, humidity, or even sterile conditions, these must be known at an early stage in the planning. The process also raises questions about the required detection limits and accuracy.

Based on the conditions mentioned, an automation concept for the metrological determination of vitamin D in blood samples was created [18]. These are individual samples that are available in 2-mL Eppendorf vials (decision criterion sample container). To avoid contamination between the patient samples, reformatting and further processing in microtiter plates are not possible (decision criterion MTP vs. single sample handling). However, to enable the greatest possible parallelization of sample processing, the samples are arranged in MTP format. The volumes to be dosed are between 10 µL and 1 mL. This enables processing on a classic liquid handling system (decision criterion liquid handling system vs. central robot). The manual sample preparation process includes the process steps of dosing, shaking, centrifugation, purification, and transfer to the measuring system. For this purpose, the corresponding devices were integrated on the deck of the liquid handler (decision criterion for sub-processes to be automated). To increase the capacity on the liquid handler deck, self-filling liquid reservoirs were integrated, which require little space on the deck and thus leave sufficient capacity for the samples (decision criterion throughput). There were no special requirements for the ambient conditions, so that additional modules for temperature or humidity control were not included. The system structure was chosen so flexibly that with minor reconfigurations, e.g., the metrological determination of THC and its derivatives from serum or urine samples is possible [19] (decision criterion flexibility).

If the sample containers are significantly larger (decision criterion sample container) and larger amounts of solvents have to be handled (decision criterion volume), classic liquid handlers can only be used to a limited extent (decision criterion liquid handler vs. central robot). This is the case, for example, when processing patient samples to isolate cells from human material. The analysis of the overall process required, among other things, the filtration of the samples, the opening, and closing of sample vessels of different dimensions (50 mL starting vessel for patient sample, 2 mL target vessel for cell pellets), as well as the automated determination of the cell pellets [25] (decision criterion sub-processes to be automated). In this case, the decision was made in favor of a system with a central robot that transports samples and labware between the subsystems for executing the sub-processes.

In addition to the process-related requirements, the available space, the investment and operating expenses, and the question of the desired flexibility must also be clarified. A high degree of flexibility required, i.e., the use of an automation system for different applications, requires an open system structure, which also enables the integration and exchange of additional modules—according to the ranges of the systems used. When establishing an automation system in the laboratory, the software must also be considered, in particular the question of what knowledge is required for programming the system. Here, there are clear differences between developers, system integrators, and end users. This also applies to external data communication such as desired data formats for the exchange (Excel, number, Adobe PDF, CSV, XML …) or the connection to LIM systems and higher-level process control systems.

### Summary and future outlooks

In conclusion, it can be stated that there is no generally valid concept for the automation of analytical and bioanalytical processes. The selection and development of a suitable system must always take place in close coordination between the user and the automation company.

A general shortcoming with all automation concepts is currently the logistics for feeding new samples and labware to the automation systems or islands and, on the other hand, the disposal of processed samples and used labware. Humans are currently used here, who take on these transport tasks between different systems. Future developments will use mobile robots that can take on such transport tasks autonomously [34]. Corresponding approaches have already been reported for life science laboratories by Liu [35] and for a chemical-synthetic laboratory by Burger [36].

Artificial intelligence will increasingly find application in the automation of (bio)analytical processes. The main area of application here is initially data analysis. AI can analyze large amounts of data and recognize patterns and trends. This can be particularly useful for evaluating large amounts of data, e.g., in medical research. (Bio)analytical methods often also require the automation of image recognition, e.g., for the detection of fill levels, phase boundaries, cell pellets, and the identification of turbidity in solutions or the detection of crystals. AI methods are increasingly being used here. However, methods of artificial intelligence can also be used to optimize process control based on measurement data and can thus take over the development and optimization of automated methods, among other things. However, AI methods can also be used to monitor quality control processes in the laboratory. For example, AI systems can be used to detect deviations from standard values in measurement processes and to take corrective measures in good time.
